# Machine Learning-Based Prediction of Elekta MLC Motion with Dosimetric Validation for Virtual Patient-Specific QA

**DOI:** 10.3390/bioengineering12121369

**Published:** 2025-12-16

**Authors:** Byung Jun Min, Gyu Sang Yoo, Seung Hoon Yoo, Won Dong Kim

**Affiliations:** 1 Department of Radiation Oncology, Chungbuk National University Hospital, Cheongju 28644, Republic of Korea; 2 Department of Radiation Oncology, Chungbuk National University College of Medicine, Cheongju 28644, Republic of Korea; 3 Proton Therapy PTE LTD, Singapore Advance Medicine, Singapore 138622, Singapore

**Keywords:** machine learning, multi-leaf collimator, Elekta Versa HD, log-file analysis, patient-specific QA

## Abstract

Accurate multi-leaf collimator (MLC) motion prediction is a prerequisite for precise dose delivery in advanced techniques such as IMRT and VMAT. Traditional patient-specific quality assurance (QA) methods remain resource-intensive and prone to physical measurement uncertainties. This study aimed to develop machine learning (ML) models to predict delivered MLC positions using kinematic parameters extracted from DICOM-RT plans for the Elekta Versa HD system. A dataset comprising 200 patient plans was constructed by pairing planned MLC positions, velocities, and accelerations with corresponding delivered values parsed from unstructured trajectory logs. Four regression models, including linear regression (LR), were trained to evaluate the deterministic nature of the Elekta servo-mechanism. LR demonstrated superior prediction accuracy, achieving the lowest mean absolute error (MAE) of 0.145 mm, empirically confirming the fundamentally linear relationship between planned and delivered trajectories. Subsequent dosimetric validation using ArcCHECK measurements on 17 clinical plans revealed that LR-corrected plans achieved statistically significant improvements in gamma passing rates, with a mean increase of 2.24% under the stringent 1%/1 mm criterion (*p* < 0.001). These results indicate that the LR model successfully captures systematic mechanical signatures, such as inertial effects. This study demonstrates that a computationally efficient LR model can accurately predict Elekta MLC performance, providing a robust foundation for implementing ML-based virtual QA. This approach is particularly valuable for time-sensitive workflows like adaptive radiotherapy (ART), as it significantly reduces reliance on physical QA resources.

## 1. Introduction

Accurate multi-leaf collimator (MLC) motion is essential for ensuring precise dose delivery in radiation therapy, particularly in advanced techniques such as IMRT and VMAT, in which dynamic leaf modulation plays a central role [1,2,3,4]. Verification of MLC performance has traditionally relied on patient-specific quality assurance (QA), log-file analysis, and mechanical testing. These methods, however, are inherently resource-intensive and reactive, thereby restricting the capability for proactive error detection prior to delivery [5].

Recent advancements in patient-specific quality assurance (QA) have increasingly utilized deep learning (DL) architectures, such as recurrent neural networks (RNNs), to capture the temporal dynamics of multi-leaf collimator (MLC) motion [1]. However, these complex models often present a “black-box” nature with high computational demands, posing challenges for clinical interpretation and real-time deployment. Furthermore, existing research has been predominantly focused on Varian linear accelerators due to the accessibility of their log files [6,7,8,9]. In contrast, Elekta systems remain underrepresented in the literature owing to their unstructured trajectory logs [10,11], with most studies limited to kinematic analysis rather than comprehensive QA plan reconstruction.

Crucially, independent of model complexity, the control systems of Elekta Versa HD (Elekta AB, Stockholm, Sweden) linacs operate on deterministic servo-mechanisms where MLC positional error is fundamentally a linear function of velocity and acceleration rather than a stochastic process. Consistent with this physical characteristic, empirical comparisons suggest that linear regression (LR) can achieve superior predictive accuracy by avoiding the overfitting to sensor noise often observed in high-variance DL models [10]. Therefore, this study adopts a physics-informed linear regression approach, prioritizing the principle of Occam’s Razor to establish a data-efficient and interpretable framework capable of immediate error detection.

The primary purpose of this study is to develop machine learning models capable of accurately predicting delivered MLC positions using parameters extracted from DICOM-RT files in Elekta Versa HD systems. To achieve this, we evaluated four distinct algorithms—linear regression, decision tree regression, bagging regression, and gradient-boosted regression—using a dataset that pairs planned leaf dynamics with actual delivery logs [12]. A key contribution of this work is the integration of these predicted trajectories into modified DICOM-RT files for downstream measurement-based gamma analysis [13,14]. This approach demonstrates the feasibility of a virtual QA framework that significantly reduces measurement workload while enhancing pretreatment verification efficiency or online adaptive therapy.

## 2. Materials and Methods

### 2.1. Data Collection and Pre-Processing

We extracted DICOM-RT plan files from the MONACO v5.11 treatment planning system (Elekta CMS, Maryland Heights, MO, USA). A total of 200 patients were randomly selected to construct an unbiased dataset. The DICOM-RT plan files contained MLC positions, gantry angles, jaw positions, and dose information, as well as non-identifying patient treatment information [15]. Log files recorded during treatment were obtained from an Elekta Versa HD LINAC. A comprehensive summary of the methodology, hardware specifications, software tools, and dataset characteristics used in this study is presented in Table 1.

Unlike the standardized XML format of Varian logs, Elekta Versa HD trajectory logs use a proprietary structure that complicates parsing; we therefore implemented a custom Python pipeline to parse these logs and align them with the DICOM-RT plan [16,17,18,19]. The pre-processing workflow comprised three steps: (1) Parsing strategy—The trajectory log from the Elekta Versa HD conforms to a proprietary, non-standard format, necessitating a dedicated parsing pipeline. We decoded the raw data stream using the open-source PyMedPhys toolkit (PyMedPhys Developers, Sydney, Australia) and extracted kinematic variables—multi-leaf collimator (MLC) positions, jaws, gantry angle, and monitor units (MU)—which were sampled at 25 Hz (40 ms intervals); (2) Synchronization—Mapping the continuous time-series recorded in the delivery log to the discrete control points (CPs) of the DICOM-RT plan is a nontrivial problem: beam-hold events and other delivery interruptions can introduce misalignments. To achieve robust synchronization, we used both time-stamps and cumulative MU, ensuring that the recorded machine state was accurately aligned with the corresponding progression of the treatment plan; and (3) Generation of matched training pairs—Applying this parsing and synchronization procedure produced a precisely matched dataset of (*X_planned_*, *X_delivered_*) pairs, which was used for subsequent model training.

**Figure 1:**
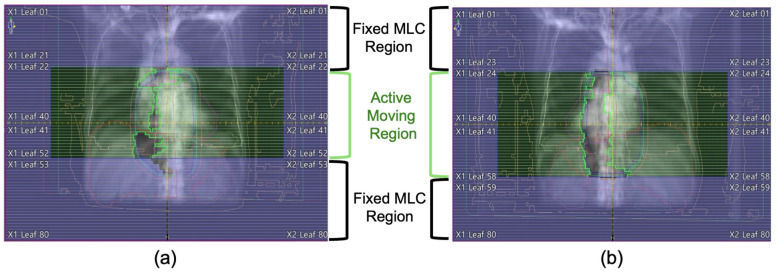
Beam’s Eye View (BEV) illustrating the dynamic modulation of the MLC aperture at two different control points obtained from two separate treatment plans. The green region represents the MLC area where modulation is activated (varying from 31 pairs in (**a**) and 35 pairs in (**b**)) and not occluded by the jaws.

As visualized in Figure 1, VMAT delivery involves dynamic aperture modulation where the set of ‘active’ leaves—those positioned within the open field defined by the jaws—changes continuously between control points. Including the large number of static, occluded leaves would introduce significant statistical bias (data sparsity), potentially causing the model to underestimate leaf motion. To mitigate this, our pre-processing algorithm was designed to dynamically isolate the active modulation region (highlighted in green) at each timestamp, extracting kinematic features exclusively from these clinically relevant leaves.

To align the datasets, the number of active MLC leaves was identified. Using extracted parameters, MLC velocity and acceleration were calculated using Equations (1) and (2):

(1) $${MLCV}_{cp}=\frac{{MLCP}_{cp}\;-{\;MLCP}_{cp-1}}{t_{cp}\;-\;t_{cp-1}}$$

(2) $${MLCA}_{cp}=\frac{{MLCV}_{cp}-{MLCV}_{cp-1}}{t_{cp}-t_{cp-1}}$$

where the subscript ‘*cp*’ denotes the sequential index of the current control point (*cp* = 1, …, N), *t_cp_* represents the cumulative timestamp, and *MLCP_cp_*, *MLCV_cp_*, and *MLCA_cp_* indicate the MLC position, velocity, and acceleration at the *cp*-th control point, respectively [10].

### 2.2. Statistical Analysis

To determine relationships among variables, aligned patient datasets were analyzed using R software (version 4.0.4; R Core Team, Vienna, Austria). MU, gantry angle, jaw position, and MLC position were selected as variables, and correlation coefficients were calculated. A preliminary linear regression (LR) analysis was conducted for a representative patient to identify variables with strong predictive potential. Statistical significance testing of the LR model was also performed [20].

### 2.3. Machine Learning Models

The scikit-learn library (version 1.2.2; scikit-learn Developers) was used to construct ML models. LR, decision-tree regression, bagging regression trees (BRT), and gradient-boosted regression trees (GBRT) were applied to predict MLC positions [12]. For the LR model, coefficients and intercepts were calculated and subsequently used to generate modified DICOM-RT plan files by substituting predicted MLC positions into the original structure. In the BRT and GBRT models, decision trees were used as the base estimators. BRT is an ensemble method that selects the best result among a set of similar trees, whereas GBRT improves performance by sequentially adding trees to correct residual errors from previous iterations.

To evaluate model performance and ensure generalization capability, the dataset was partitioned into training (70%), validation (15%), and test (15%) sets. The validation set was utilized for hyperparameter optimization and early stopping, while the test set was strictly reserved for the final performance assessment of the trained models. To mitigate potential selection bias associated with a single random split and to prevent data leakage arising from the high temporal autocorrelation of adjacent control points, this procedure was executed using a 5-fold cross-validation scheme performed on a patient-wise basis. This ensures that all control points from a single patient or treatment arc were assigned exclusively to either the training, validation, or testing sets. Consequently, the evaluation metrics (MAE) represent the model’s performance on entirely unseen clinical cases, validating its generalization capability beyond the training data. Input features included MLC position, velocity, and acceleration extracted from DICOM-RT plan files. The target labels were the corresponding delivered MLC positions extracted from log files. In addition, the MLC opening area (field of view, FOV) was computed from both the plan and log files using Equation (3):

(3) $$FOV=\sum_{n=start}^{end}\left(X_{1}(n)-X_{2}(n)\right)$$

where *n* denotes the MLC leaf number, *X*_1_ the left-leaf position, and *X*_2_ the right-leaf position.

Model performance was evaluated using the mean absolute error (MAE), calculated as:

(4) $$MAE=\frac{1}{n}\sum_{i=1}^{n}\left({Data}_{DICOM}(i)-{Data}_{Prediction}(i)\right)$$

Predicted parameters were imported back into MONACO v5.11 to compute dose distributions and generate modified DICOM-RT plan files, which were subsequently exported to the LINAC for measurement.

### 2.4. Gamma Passing Rate for Patient QA

**Figure 2:**
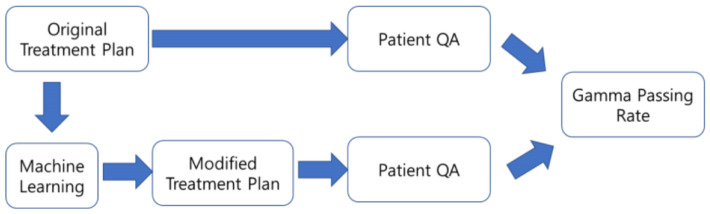
Schematic process of patient-specific QA and gamma passing rate evaluation with original treatment plan and modified treatment plan using a machine learning algorithm.

ML-predicted MLC parameters were used to generate modified DICOM-RT plan files, as illustrated in Figure 2. Among the models evaluated in Section 2.3, the algorithm with the lowest error was selected, and its predicted parameters were converted into a clinically recognizable DICOM-RT format using an in-house conversion tool.

To validate the clinical deliverability and dosimetric accuracy of the ML-predicted MLC trajectories, physical measurements were performed using the ArcCHECK diode array (Sun Nuclear, Melbourne, FL, USA). ArcCHECK was selected as the reference dosimeter due to its capability for 3D isotropic measurement, which is considered a gold standard for verifying rotational VMAT deliveries. This physical validation serves as a ground truth to confirm that the discrepancies predicted by the ML model translate into actual dosimetric improvements.

Seventeen patient cases were selected for end-to-end testing to evaluate patient-specific QA performance. Measurements were performed on the same day using both the original and ML-predicted DICOM-RT plans with an ArcCHECK device. Gamma passing rates were calculated using both 2%/2 mm and 1%/1 mm criteria [21].

## 3. Results and Discussion

### 3.1. Statistical Analysis

The statistical relationships between variables extracted from the DICOM-RT plan files and Elekta log files are summarized in Table 2. The correlation coefficients for corresponding parameters in the plan and log data were approximately 1.0, confirming strong linear consistency between planned and delivered machine parameters. Monitor unit (MU) showed low correlation (<0.2) with gantry angle and jaw positions, suggesting these variables are largely independent. In contrast, MLC positional variables exhibited moderate correlation with MU (>0.5), consistent with previous findings that delayed or inaccurate MLC motion can influence delivered dose by altering the field-of-view (FOV) through which radiation is transmitted.

Linear regression (LR) analysis performed on a representative case revealed slope coefficients close to 1.0 and intercepts < 0.1 (Table 3), indicating that delivered MLC positions closely follow planned trajectories. The non-zero regression coefficients and statistically significant *p*-values (*p* < 0.001) support meaningful linear associations between variables [20]. These results guided the selection of ML models and the construction of variable sets for prediction tasks.

### 3.2. Model Performance

Across all evaluated ML models, LR consistently demonstrated the highest prediction accuracy for delivered MLC positions. As shown in Table 4 and Table 5, LR achieved the lowest mean absolute error MAE = 0.145 mm and 0.199 mm, respectively. Tree-based models, including DT, BRT, and GBRT, exhibited higher prediction errors, particularly for leaves undergoing large positional excursions. Although GBRT improved stability compared with single-tree approaches, it did not surpass LR in predictive accuracy.

The linear regression model yielded a coefficient of determination (R^2^) of 0.308. While a high R^2^ is typically desired, this metric must be interpreted within the context of the intrinsic characteristics of the Elekta Versa HD’s high-precision servo system. Crucially, the target variable in this study is the minute positional error rather than the macroscopic leaf position. Consequently, the total variance (*SS_tot_*) is dominated by stochastic sensor noise comparable in magnitude to the systematic deviations, creating a low signal-to-noise ratio (SNR) regime where R^2^ is inherently suppressed. Notably, the ‘unmodeled variability’ highlighted by the low R^2^ corresponds largely to this irreducible random noise. This is evidenced by the fact that complex non-linear models (e.g., decision trees) attempted to fit this noise, resulting in negative R^2^ values indicative of overfitting. In contrast, the linear regression model successfully decoupled the systematic mechanical signatures (e.g., lag and gravity effects) from the stochastic background. Therefore, the validity of the model is best evidenced not by statistical correlation, but by its downstream clinical impact: an MAE of 0.145 mm and a statistically significant improvement in Gamma Passing Rates (+2.24%, *p* < 0.001). This confirms that the portion of variance explained by the model corresponds precisely to the deterministic errors affecting clinical dose delivery.

Prediction accuracy was reduced when only MLC position was used as the input, compared to models using position, velocity, and acceleration. Chuang et al. also reported that LR models using velocity alone performed worse than more comprehensive models [9,10,11]. Additional variables may therefore influence MLC positional errors in regression-based prediction. The MAE and coefficient of determination for each model are summarized in Table 4. LR achieved the lowest MAE, while tree-based models produced MAE values between 0.20 and 0.24 mm. gradient-boosted regression improved stability over single-tree models but did not exceed LR in accuracy.

The models were further evaluated using FOV-based prediction metrics, where the aperture opening (computed from opposing leaf pairs) served as the target variable. Table 6 shows that multivariate LR again achieved the lowest MAE, outperforming DT, BRT, and regularized LR variants (lasso, ridge). Tree-based models showed elevated errors, particularly in control points with rapid modulation of the radiation field. The superior performance of LR indicates that MLC aperture dynamics also follow a primarily linear relationship with planned openings. Regularization methods produced intermediate performance, suggesting that excessive coefficient shrinkage may hinder the model’s capacity to capture deterministic changes in beam aperture. Collectively, these results confirm that both leaf-level and aperture-level MLC behaviors are best reconstructed using LR-based models.

Five-fold stratified cross-validation demonstrated consistent LR performance across all folds, with a standard deviation of only 0.008 mm in MAE. This confirms that the LR model generalizes well to unseen control points and is not susceptible to overfitting, even with variability in gantry rotation speed or leaf motion complexity.

Among the tested algorithms, linear regression (LR) consistently achieved the highest prediction accuracy, yielding the lowest MAE across leaf-level and field-of-view evaluations. The superiority of LR suggests that Elekta’s MLC trajectories are predominantly governed by linear mechanical behavior, likely reflecting the deterministic control structure of the Elekta MLC servo-drive system. This finding is consistent with prior work reporting strong linear dependencies in MLC positional dynamics. Importantly, LR also showed minimal variability in k-fold cross-validation, indicating robust generalization despite moderate dataset size. Given its computational efficiency and interpretability, LR is an attractive candidate for real-time or near–real-time prediction and for workflow-integrated quality-assurance (QA) applications.

While advanced non-linear models such as neural networks or gradient boosting are powerful tools for complex pattern recognition, our comparative analysis demonstrated that linear regression (LR) outperformed these complex architectures in predicting Elekta MLC positions [10,22]. This counter-intuitive finding is attributed to the deterministic nature of the Elekta servo-control mechanism, where positional errors exhibit a predominantly linear dependence on kinematic parameters (velocity and acceleration). In this high-precision, low-variance domain, complex models tended to overfit to random log-file noise, whereas LR robustly captured the underlying mechanical trend. Therefore, the choice of LR is justified not only by its computational efficiency for real-time QA but also by its superior empirical accuracy in this specific physical context.

### 3.3. Dosimetric Verification and Gamma Passing Rate Analysis

Dosimetric validation was performed using an ArcCHECK device across 17 clinical cases. Gamma passing rate results (2%/2 mm and 1%/1 mm) for both original and ML-predicted DICOM-RT plans are summarized in Table 7 and Table 8. ML-generated MLC predictions produced lower MAE values than the discrepancies between planned and delivered MLC positions. The LR and GBRT models achieved the highest overall accuracy, and in this experiment, the predicted plan using the LR model was employed.

For the 2%/2 mm criterion, 12 of 17 plans demonstrated improved gamma performance with predicted trajectories, with a mean improvement of 0.46%. The paired *t*-test yielded a statistically significant *p*-value of 0.0458 (95% CI: 0.008–0.909). Under the more stringent 1%/1 mm criterion, all 17 plans showed improvement, with an average gamma increase of 2.24% (95% CI: 1.718–2.752, *p* < 0.001).

A gamma pass rate below 100% indicates discrepancies between the delivered and the planned MLC motions. Even when pass rates exceed 95%, further improvement toward 100% is both dosimetrically and clinically meaningful, as it reduces residual delivery uncertainties and improves the fidelity of dose delivery. Given that modern LINACs and planning systems already achieve high passing rates (>95%) under standard criteria, the margin for numeric improvement is intrinsically limited. The substantive clinical impact is demonstrated under the stricter 1%/1 mm criterion, where the model yielded a mean improvement of 2.24% (*p* < 0.001). In the context of high-precision radiotherapy (SRS/SBRT), where plans are more sensitive to mechanical latency and often yield marginal QA results, a 2.24% enhancement is non-trivial. It effectively bridges the gap between the idealized treatment planning system (TPS) calculation and the actual machine delivery. The proposed virtual QA framework enables rapid verification of treatment plans without physical QA by more accurately predicting these discrepancies.

It is important to note that while the planned (input) and delivered (label) MLC positions exhibit high correlation due to the precision of the control system, they are not identical [13]. The minimal yet clinically significant discrepancies arise from mechanical latency and gravitational effects. Our model does not merely replicate the input parameters; rather, it identifies and predicts the systematic residuals—the ‘mechanical signature’ of the specific machine. The validity of this learning is evidenced by the significant improvement in gamma passing rates (Table 7 and Table 8), which would not have occurred if the model functioned solely as an identity mapping.

A critical component of this study was the integration of predicted MLC trajectories into modified DICOM-RT plan files and subsequent dosimetric verification. All modified plans were confirmed to be structurally deliverable. This feasibility was explicitly verified through a two-step validation process. First, kinematic checks were applied to ensure that the predicted leaf velocities remained within the Elekta Agility limit of 3.5 cm/s and that no leaf crossovers (collisions) occurred. Second, the structural integrity of the reconstructed DICOM-RT files was validated by importing them back into the treatment planning system (TPS) and successfully executing a full dose recalculation without error flags, confirming compatibility with the machine’s control logic. Gamma analyses using stringent criteria (2%/2 mm and 1%/1 mm) consistently demonstrated improved or comparable performance relative to the original plans. These results indicate that the predicted trajectories preserve clinically acceptable dosimetric characteristics and further support the feasibility of machine learning-based virtual QA. While virtual QA is not intended to fully replace conventional patient-specific QA, it provides meaningful complementary value: (1) early detection of potential mechanical or planning inconsistencies, (2) reduction of QA measurement burden, and (3) reduction of scheduling conflicts due to limited LINAC availability for QA. Such benefits are particularly relevant in high-volume clinical environments.

The scope of this study is within the broader landscape of virtual QA, which includes Monte Carlo simulations, deep learning (DL), and analytical modeling [16,19]. While Monte Carlo methods provide high dosimetric accuracy, their substantial computational cost often hinders their application in time-sensitive workflows. Similarly, complex DL architectures may introduce latency and infrastructure requirements (e.g., high-end GPUs) that complicate clinical deployment. Since the primary objective of this work is to establish a framework applicable to the online adaptive radiotherapy (ART)—where rapid calculation is paramount—we deliberately focused on lightweight Machine Learning algorithms. Therefore, comprehensive comparisons with computationally intensive methods (e.g., Monte Carlo) were considered outside the scope of this study. Instead, we prioritized identifying the most accurate model among fast-execution algorithms (Linear Regression and Tree-based models) capable of providing immediate feedback while the patient is on the treatment couch.

It is noteworthy that while Elekta MLC motion is predominantly linear, a machine learning (ML) framework offers distinct advantages over simple deterministic mechanical corrections (e.g., fixed latency shifts). A simple deterministic model often struggles to simultaneously optimize multiple kinematic factors—such as velocity, acceleration, and gravitational effects—without manual fine-tuning. In contrast, the Linear Regression model functions as a data-driven multivariate optimizer, automatically determining the precise coefficients that minimize residual errors across thousands of control points. Furthermore, this ML-based approach provides adaptability; the model can be periodically retrained to track temporal changes in machine performance due to wear and tear, ensuring long-term robustness that a static mathematical formula cannot guarantee.

While this study focused on verifying plans under normal operating conditions, the proposed ML framework provides a robust mechanism for machine health monitoring and anomaly detection. By establishing a baseline for normal delivery, the Linear Regression model allows acute anomalies, such as motor failures, to be flagged via significant residuals, while periodic retraining enables the tracking of calibration drift and machine aging through gradual changes in learned coefficients (*w_vel_*, *w_acc_*). Furthermore, although the model approximates non-linear behaviors like backlash using velocity and acceleration terms, any unmodeled mechanical looseness manifests as increased prediction error (MAE), thereby serving as a diagnostic indicator that alerts physicists to the need for mechanical adjustments.

The primary limitation of this study is the exclusive reliance on data sourced from a single Elekta Versa HD system. Given the known inter-machine variability in MLC calibration, motor friction, and control behavior across different institutions, external validation using multi-institutional datasets and diverse treatment cohorts remains essential to establish broader applicability. Furthermore, the current dataset size did not permit a detailed analysis of rare MLC anomalies, which may affect predictive performance in outlier scenarios. Although k-fold cross-validation was rigorously implemented to minimize internal bias associated with the limited data, this limitation must be addressed externally. Nevertheless, the observed efficacy of the Linear Regression model is fundamentally rooted in the control architecture of the Elekta Agility head, which utilizes an optical feedback loop highly conducive to linear kinematic modeling. We therefore propose that the framework is generalizable via local adaptation. While the simple linear model structure remains valid across different Elekta machines, the learned coefficients serve as machine-specific signatures. Implementing this method at another institution thus requires local retraining rather than a new algorithm, allowing the linear equation to recalibrate its slopes to the specific wear characteristics of the target machine, thereby ensuring robust performance independent of device age or calibration history.

Although this study relies on data from a single institution, the rigorous cross-validation mitigates potential bias. However, external validation on diverse machine configurations remains necessary. To provide a comprehensive overview of the proposed framework’s clinical utility and future directions, a SWOT analysis is summarized in Table 9.

Despite these limitations, the present work makes a meaningful contribution to the radiotherapy QA field. The results presented here provide valuable insight and a foundation for Elekta-specific or vendor-agnostic predictive QA frameworks [22,23]. The demonstrated feasibility of generating clinically deliverable, dosimetrically acceptable predicted plans further highlights the potential of ML-driven tools to enhance safety, efficiency, and precision in modern radiotherapy workflows. The demonstrated feasibility of generating clinically deliverable, dosimetrically acceptable predicted plans further highlights the potential of ML-driven tools to enhance safety, efficiency, and precision in modern radiotherapy workflows.

## 4. Conclusions

This study demonstrated that MLC leaf positions in an Elekta VersaHD system can be accurately predicted using machine learning models trained on treatment planning parameters and log-file data, with linear regression providing the most reliable performance due to the predominantly linear nature of Elekta MLC motion. Predicted trajectories were successfully embedded into modified DICOM-RT plans and produced dose distributions that met stringent gamma analysis criteria, confirming their clinical feasibility. These findings highlight the value of machine learning-based prediction as an efficient supplement to conventional QA, offering potential benefits in early error detection and reduced measurement workload. Although limited to a single LINAC and institution, this work establishes a practical foundation for broader multi-institutional development and future integration of data-driven virtual QA into clinical workflows.

In summary, this work establishes a practical and effective approach for predicting Elekta MLC behavior using machine learning methods and underscores the potential of data-driven virtual QA to strengthen safety and efficiency in modern radiotherapy practice or online adaptive radiotherapy.

## Figures and Tables

**Table 1:** Summary of materials, tools, and methodologies used in the study.

Category	Item	Specification/Description
Hardware	Linear Accelerator	Elekta Versa HD™ with Agility™ MLC (160 leaves)
	QA Device	Sun Nuclear ArcCHECK^®^ (Cylindrical Diode Array)
Software and Tools	Treatment Planning	MONACO v5.11 (Elekta CMS)
	Programming Environment	Python (v3.11) for ML, R (v4.0.4) for Statistics
	Key Libraries	PyMedPhys v0.39 (Log analysis), scikit-learn v1.2.2 (Model training)
Dataset	Data Source	200 VMAT plans (DICOM-RT) paired with Trajectory Logs
	Input Features	Planned MLC Position (x), Velocity (v), Acceleration (a)
	Target Variable	Delivered MLC Position (Log-recorded)
Modeling	Algorithms Evaluated	Linear Regression (LR), Decision Tree (DT), Bagging, GBRT
	Validation Method	5-fold Stratified Cross-Validation (Patient-wise split)
Evaluation	Statistical Metrics	Mean Absolute Error (MAE), R^2^
	Dosimetric Metrics	Gamma Passing Rate (Criteria: 2%/2 mm, 1%/1 mm)

**Table 2:** Pearson correlation coefficients between kinematic parameters extracted from DICOM-RT plans and Elekta trajectory Logs.

Parameters	D. MU	T. MU	D. GA	T. GA	D. Y	T. Y	D. X	T. X
D. MU	**1**	**0.999**	−0.006	−0.008	−0.013	−0.013	**0.546**	**0.545**
T. MU	**0.999**	**1**	−0.006	−0.008	−0.013	−0.013	**0.546**	**0.545**
D. GA	−0.006	−0.006	**1**	**0.989**	−0.127	−0.012	0.117	0.113
T. GA	−0.008	−0.008	**0.989**	**1**	−0.129	−0.012	0.116	0.112
D. Y	−0.013	−0.013	−0.127	−0.129	**1**	**0.999**	−0.078	−0.068
T. Y	−0.013	−0.013	−0.127	−0.129	**0.999**	**1**	−0.078	−0.067
D. X	**0.546**	**0.546**	0.117	0.116	−0.078	−0.078	**1**	**0.999**
T. X	**0.545**	**0.545**	0.113	0.112	−0.068	−0.067	**0.999**	1

Abbreviations: D. = Parameters extracted from DICOM-RT plan (Planned values); T. = Parameters extracted from Elekta trajectory log (Delivered values); MU = Monitor unit; GA = gantry angle; Y = jaw position; X = MLC position. Note: Coefficients with an absolute value greater than 0.5 are highlighted in bold to indicate moderate-to-strong correlations.

**Table 3:** Results of Linear Regression.

	Coefficient	Intercept	*p*-Value
MU	0.999	−0.146	1.58 × 10^−8^
Gantry Angle	0.989	0.186	5.37 × 10^−16^
Y1 Jaw	0.997	0.214	2.00 × 10^−16^
Y2 Jaw	0.998	−0.157	2.00 × 10^−16^
X1 MLC	0.993	−0.167	2.00 × 10^−16^
X2 MLC	0.996	0.066	2.00 × 10^−16^

**Table 4:** MAE, R^2^ of ML with position, velocity, and acceleration in MLC.

Models	MAE	R^2^
Linear Regression	0.145	0.308
Decision Tree Regression	0.172	−0.109
Bagging Regression Tree	0.16	0.187
Gradient Boosted Regression Tree	0.146	0.272
MLC Positional Errors	0.22	

**Table 5:** MAE, R^2^ of ML with the only positions of MLC.

Models	MAE	R^2^
Linear Regression	0.199	−0.002
Decision Tree Regression	0.236	−0.373
Bagging Regression Tree	0.222	−0.22
Gradient Boosted Regression Tree	0.2	−0.044
MLC Positional Errors	0.22	

**Table 6:** MAE of ML with field of view (FOV) of MLC.

Models	MAE
Linear Regression	1.49
Multivariate Linear Regression	7.27
Decision Tree Regression	149.33
Bagging Regression (Lasso)	7.79
Bagging Regression (Ridge)	7.56
The actual FOV Errors	28.26

**Table 7:** Comparison of gamma passing rates (2%/2 mm) between the original plan and the ML-predicted plan for each Patient Number (PN).

PN	1	2	3	4	5	6	7	8	9	10	11	12	13	14	15	16	17
OP	92.2	96.8	96.8	94.9	93.7	97.3	95.6	91.7	97.8	97.6	89.8	94.1	95.5	96.3	96.1	96.6	95.4
PP	92.8	97.5	97.1	97.7	95.7	97.9	95.9	92.5	98	97.5	89.5	94.5	95.8	96.9	96	95.5	95.2

PN: Patient Number, OP: Original Plan, PP: Predicted Plan.

**Table 8:** Comparison of gamma passing rates (1%/1 mm) between the original plan and the ML-predicted plan for each Patient Number (PN).

PN	1	2	3	4	5	6	7	8	9	10	11	12	13	14	15	16	17
OP	86.8	94.3	93.1	94.4	91	95.7	89.2	93.1	95.3	88.8	93.7	85.9	88.4	95.8	89.3	89.9	86.1
PP	90.6	96.6	95.3	95.3	93	97.2	92.4	93.4	96.1	91	96.1	88.8	90.5	97.5	92.5	92.5	90

PN: Patient Number, OP: Original Plan, PP: Predicted Plan.

**Table 9:** SWOT analysis of the machine learning-based Virtual QA framework for Elekta MLCs.

Strengths	Weaknesses
High Efficiency: Low computational cost enables real-time verification suitable for clinical routine.	Single-Institution Data: Model generalization to other machines requires further validation.
Interpretability: Linear models align with the deterministic nature of Elekta servo-control.	Linear Constraint: May not capture rare, complex, non-linear mechanical anomalies.
Data Efficiency: Robust performance even with smaller datasets compared to Deep Learning.	Prototype Status: Lack of a fully integrated graphical user interface (GUI).
Opportunities	Threats
Adaptive Radiotherapy (ART): Critical for time-sensitive QA in online adaptive workflows.	Log-File Dependency: Reliability is contingent on the integrity of the machine’s internal sensors.
Multi-Center Expansion: Potential for cloud-based or federated learning applications.	Vendor Updates: Vulnerability to changes in proprietary log formats or encryption.
Complementary Tool: Acts as an independent “second check” alongside physical dosimetry.	Regulatory Standards: Current protocols (TG-218) still mandate physical measurement.

## Data Availability

The datasets presented in this article are not readily available due to institutional restrictions. Requests to access the datasets should be directed to Correspondence.

## Abbreviations

The following abbreviations are used in this manuscript:

BRT	Bagging Regression Tree
CP	Control Point
DICOM-RT	Digital Imaging and Communications in Medicine for Radiation Therapy
DT	Decision Tree Regression
FOV	Field of View
GBRT	Gradient-Boosted Regression Tree
IMRT	Intensity-Modulated Radiation Therapy
LINAC	Linear Accelerator
LR	Linear Regression
MAE	Mean Absolute Error
ML	Machine Learning
MLC	Multi-Leaf Collimator
MLCA	MLC Acceleration
MLCP	MLC Position
MLCV	MLC Velocity
MU	Monitor Unit
QA	Quality Assurance
VMAT	Volumetric Modulated Arc Therapy
